# A Novel Tool for Collaborative and Blinded Orthopedic Image Analysis

**DOI:** 10.3390/life13091805

**Published:** 2023-08-24

**Authors:** Philipp Schippers, Andrea Meurer, Matthias Schnetz, Larissa Ewald, Christian Ruckes, Reinhard Hoffmann, Yves Gramlich

**Affiliations:** 1 Department of Orthopedic Surgery, University Medical Centre, Johannes Gutenberg University Mainz, 55131 Mainz, Germany; 2 Medical Park Kliniken Bad Wiessee, 83707 Bad Wiessee, Germany; a.meurer@medicalpark.de; 3 BG Unfallklinik Frankfurt am Main gGmbH, 60389 Frankfurt am Main, Germanylarissa.ewald@bgu-frankfurt.de (L.E.); yves.gramlich@bgu-frankfurt.de (Y.G.); 4 Interdisciplinary Centre for Clinical Trials Mainz, University Medical Center, Johannes Gutenberg University Mainz, 55131 Mainz, Germany; ruckes@izks-mainz.de

**Keywords:** Tyche, collaborative image analysis, blinded image analysis

## Abstract

Image analysis plays a central role in orthopedics and research but comes with many challenges, including anonymization, bias, and achieving efficient analyses using multiple independent observers. Appropriate software is still lacking. Tyche is a free online tool that displays images in a random order without showing any metadata. Additionally, when using Tyche, observers can store results in the same window, and the final results are immediately visible to the project manager. In this study, we compared results from Tyche with those from a validated tool. One hundred pelvic radiographs were analyzed separately by five orthopedic surgeons using both Tyche and the validated software. Common orthopedic measurement modalities and scores were determined. The methods were compared using intra-class correlations and Fleiss’ kappa coefficients as well as Bland–Altman plots. Significant correlations ranging from r = 0.17 (Kallgren and Lawrence Score) to r = 0.99 (area measurements) were calculated for inter- and intraobserver agreements between the two tools for all measurements. The Bland–Altman plots indicated the non-inferiority of either tool. The images were analyzed significantly faster when Tyche was used. We conclude that Tyche is a valid tool for use in orthopedic image analysis. Tyche could be utilized for determining inter- and intraobserver agreements, in multicenter studies and for score validations.

## 1. Introduction

### 1.1. Challenges in Image Analysis

Image analysis plays a central role in orthopedics and orthopedic research in which multiple experts assess images daily to find patterns and correlations and support or dismiss hypotheses. The information gathered from these analyses determines the outcomes of decision making and treatment algorithms and has a massive impact on patients. In recent decades, tremendous progress has been achieved with respect to imaging quality in radiology and the implementation of new technologies such as convolutional neural networks [1,2]. However, the analyses performed by advanced machine learning algorithms can be biased [3,4,5]. Thus, objective assessments performed by multiple blinded experts remain vital.

When performing image assessments, challenges such as subjectivity and bias can occur. Radiologic images include vast sets of metadata such as patient names, dates of birth, and dates of acquisition. These data are mostly visible by default in the Picture Archiving and Communication System (PACS) or other Digital Imaging and Communications in Medicine (DICOM) viewers and may thus potentially influence the analysis [6,7]. When opening images from a desktop folder, one can usually read the file name and may already be able to deduct some information, such as the group name or acquisition date. For this reason, Nature, for instance, requires authors to declare if and how randomization was performed and whether data analyses were carried out blinded; if not, the authors must provide a reason [8].

Another challenge is the collaborative aspect, for instance, in the context of multicenter studies and research [9]. Involving multiple experts requires that data or images, which are accompanied by a spreadsheet with questions and a template for observers to note their results, to be shared via e-mail, USB sticks, or cloud services. Finally, all these data must be merged, which can be a significant source of calculation and transmission errors [10,11].

### 1.2. Available Tools

There are many tools and methods that can be used to analyze images [12]. Here, the focus was on radiologic images, which are often viewed and analyzed inside a hospital’s PACS viewer. These provide basic utils such as length and angle tools. More complex software, such as mediCAD^®^ v6.1 (mediCAD Hectec GmbH, Altdorf, Germany), allows for sophisticated measurements and orthopedic planning, such as knee replacements [13]. However, these tools have limited capacities for anonymization, are desktop-based, and are not well-suited for analyses by multiple observers. To include multiple observers, survey tools such as SurveyMonkey^®^ (SurveyMonkey, San Mateo, CA, USA) could be employed, yet survey tools have limited means of displaying scientific or radiologic images and usually do not provide tools for analyzing them. Instead, they are suited to gathering opinions [14] and are often used for quality management or collecting patient-reported outcome measures (PROMs), such as the functions provided by Heartbeat^®^ (Heartbeat Medical Solutions GmbH, Berlin, Germany). Blinder v1.2.0 (Steven Cothren, Solibyte Solutions LLC, Durham, NC, USA) is a desktop-bound software that displays images in a random order next to a definable score; thus, it can be seen as a significant step toward simplifying anonymized and blinded image scoring. However, Blinder does not display medical images in DICOM format or videos, it does not include tools for performing measurements, and cannot include observers located in different places. Thus, Blinder lacks the collaborative aspect of image analysis. Finally, Blinder (as of this writing) was last updated in 2019 and only works on Windows^®^ operating systems [15]. A collaborative aspect is provided by annotation tools such as Redbrick.ai (Zantula, Inc., Claymont, DE, USA) or Zillin.io (Adaptive Vision, Gliwice, Poland). Such tools allow experts to annotate images online. These annotations can then be used to train a machine learning algorithm, which is the primary purpose of these tools.

### 1.3. Tyche

Since none of the available tools addresses the above-mentioned challenges in image analysis, this study aimed to investigate a recently introduced browser-based program named Tyche™ (©Philipp Schippers, Mainz, Germany). Tyche displays scientific images inside any modern web browser in random order without showing any metadata such as the file name, date of acquisition, or patient-specific details. In addition, Tyche provides a means of storing the results in the same window in customizable input forms. Collaborative analyses are simplified by providing temporary access via uniform resource locators (URLs). Finally, the results are immediately merged and calculated and are visible online. The name of this program, Tyche, is based on the ancient Greek goddess of destiny and luck [16]. It must be noted that Tyche does not perform any automated analysis itself.

Tyche has already been successfully used in several peer-reviewed studies. Schippers et al. used Tyche to define normal and abnormal alignments for the second to fifth metatarsophalangeal joints and correlated their results with angle measurements [17]. Seitaj et al. employed Tyche to allow different observers to score mitochondrial shapes [18]. Finally, Jain et al. used Tyche to allow multiple observers to count specifically stained cells derived from fluorescence microscopy [19]. However, thus far, no validation study has been performed for Tyche. Hence, this study aimed to test and validate Tyche by comparing results from Tyche with those from a validated, commercially available orthopedic planning tool, mediCAD^®^ v6.1.

## 2. Material and Methods

### 2.1. Tyche Set Up

Tyche was set up as a browser-based program running on a Linux server. It employs Joomla!^®^ (Open Source Matters, Inc., Joomla community, New York, NY, USA) for user management and several publicly licensed JavaScript libraries: dropzone (Matias Meno, MIT License) is used to upload images, and cornerstone (Chris Hafey, MIT License) and cornerstone Tools (Chris Hafey, MIT License) are used to display, analyze, and annotate DICOM images. Its programming languages are PHP (the PHP development team, Zend Technologies) and JavaScript^®^ (Oracle Corporation, Austin, TX, USA), as well as *MySQL* (Oracle Corporation, Austin, TX, USA) for database management. All connections are secure sockets layer (SSL)-encrypted.

### 2.2. Statistics

This study compared a new tool (Tyche) with an established tool, mediCAD^®^ (mediCAD Hectec GmbH, Altdorf, Germany). To validate Tyche and its workflow, standard measuring modalities employed in radiology and orthopedics were chosen: length, angle, area, and a score. To compare the two methods/tools, the Blond–Altman plot proved to be a useful statistical approach [20,21]. Defining an alpha of less than 0.05 and a beta of more than 0.2 and expecting a mean difference of 0, the maximum allowed difference between the methods was set to 2.53 standard agreement limits. Hence, a sample size of 98 was calculated and rounded up to 100 images to be analyzed by 5 analyzers [22].

For inter- and intraobserver reliability, intra-class correlation (ICC) coefficients were calculated for metric measurements (length, angle, and area) and interpreted according to Koo and Li [23], as shown in Table 1. For the non-metric assessments, Fleiss’ kappa coefficients were calculated and interpreted according to Landis and Koch [24], as shown in Table 1.

### 2.3. Study Design

**Figure 1:**
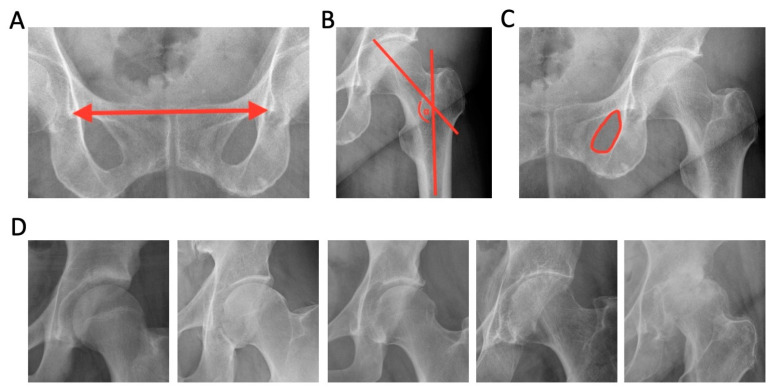
Parameters that were measured using mediCAD^®^ and Tyche. (**A**) (length): the distance between the Köhler teardrops; (**B**) (angle): the collum–center–diaphysis angle; (**C**) (area): the left obturator foramen; (**D**) (score): the Kellgren and Lawrence score, with sample images for grades 0, 1, 2, 3, and 4, defined as follows: grade 0 (none) = a definite absence of changes in osteoarthritis on an X-ray image; grade 1 (doubtful) = doubtful joint space narrowing and possible osteophytic lipping; grade 2 (minimal) = definite osteophytes and possible joint space narrowing; grade 3 (moderate) = moderate multiple osteophytes, definite narrowing of the joint space, some sclerosis, and possible deformity of bone ends; grade 4 (severe) = large osteophytes, marked narrowing of the joint space, severe sclerosis, and definite deformity of bone ends.

The most recent one hundred anteroposterior (ap)-view pelvic radiographs from our database were selected, anonymized, and exported using Advanced REVIEW Station (Dedalus Healthcare Group, Bonn, Deutschland). They were stored as DICOM files and named chronologically from “1” to “100”. Images with unique findings, such as rare pathologies or piercings that could reveal the patient’s identity, were excluded. The following parameters were determined: (A) length, the distance between the Köhler teardrops; (B) angle, the left collum–center–diaphysis angle (CCD angle), also referred to as the neck-shaft angle (NS angle); (C) area, the left obturator foramen; and (D) score, the Kellgren and Lawrence score (KLS) for the left hip (Figure 1).

**Figure 2:**
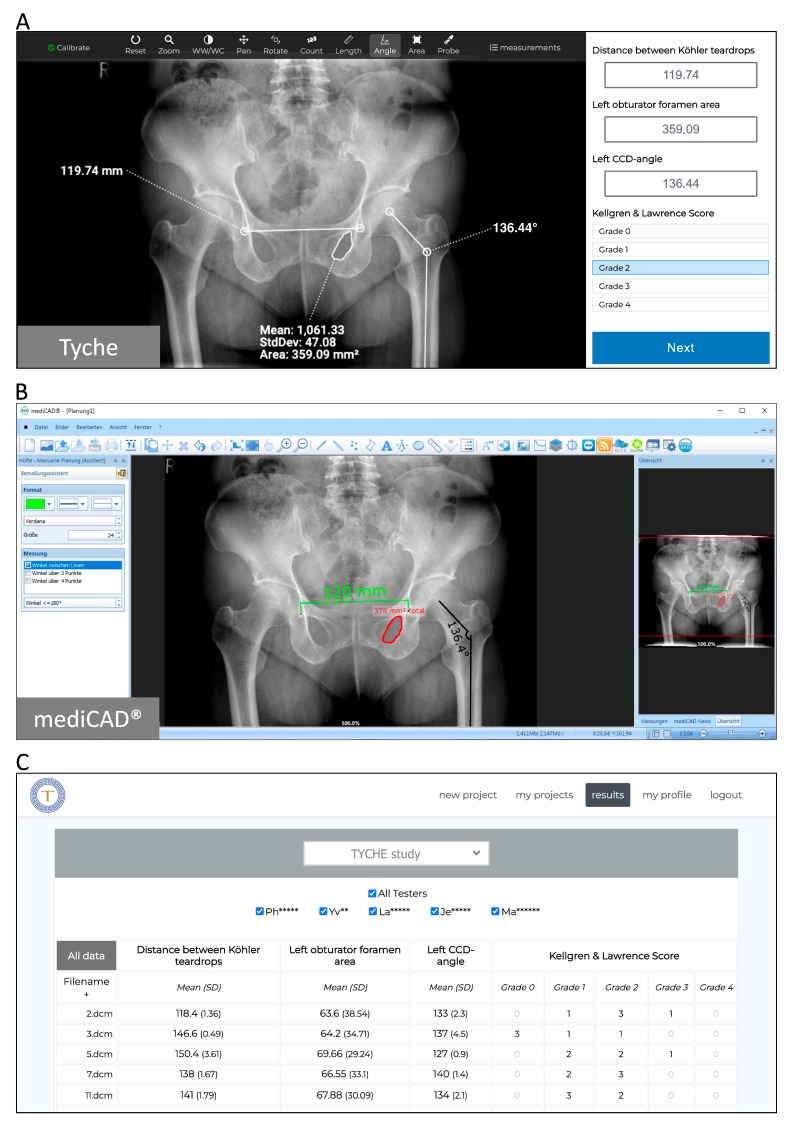
Image analysis and immediate visibility of results, as seen when using Tyche in a web browser. (**A**) Shows the core functions of Tyche, which can be seen when analyzing images inside a web browser. On the left side, an anonymized and randomly selected image from the database is shown. A set of tools is provided on top. The right side shows the questions and answers defined by the project master that must be answered by the analyzers for every single image. (**B**) Shows the same image being analyzed by analyzers using mediCAD^®^. When using mediCAD^®^, the results were noted on a separate spreadsheet. No Excel or datasheet-based protocolling was available. (**C**) Shows the results that are visible to the project master immediately after an analyzer finishes performing the analysis using Tyche. Every row represents an image; the first column shows the file name, and the following columns show the results for the questions shown in the headline of each column.

To analyze the images using Tyche, the following steps were taken: (I) A project name was chosen. (II) Numeric questions were created for length, angle, and area measurements. For the KLS, a single-choice question with five possible answers (grades 0 to 4) was created. (III) The images were uploaded via drag-and-drop. To initialize the analysis, observers were invited to participate in the analysis via an automatically created project-specific URL, which was only temporarily valid. The observers used a web browser to view the images randomly, without knowing the image name and without any metadata. Next to each image, the above-defined questions with answers were shown and answered using the measuring tools on top of each image (Figure 2A). After an observer finished the analysis, the results were immediately visible online to the project manager (Figure 2C).

For the image analysis with mediCAD^®^, the images were opened one by one, and the corresponding measurement tools were selected (Figure 2B). Results were noted on a separate spreadsheet.

For the length measurements, using both Tyche and mediCAD^®^, the images were calibrated to 100%. In mediCAD^®^, this was achieved via the calibration tool; calibration is mandatory before performing length measurements. Using Tyche, the calibration tool, which can be found in the top left corner (Figure 2A), was used. Tyche permits length measurements without calibration; however, in this case, the units are shown in pixels (“px”) instead of millimeters (“mm”). In both tools, a calibration factor of “1” was entered.

To estimate the time needed by one observer to analyze one image, the observers were asked to note the time required to analyze twenty images using mediCAD^®^, and the total time was divided by twenty to calculate the necessary time for one image. In Tyche, the time needed to analyze one image was deducible from automatically stored log files.

**Figure 3:**
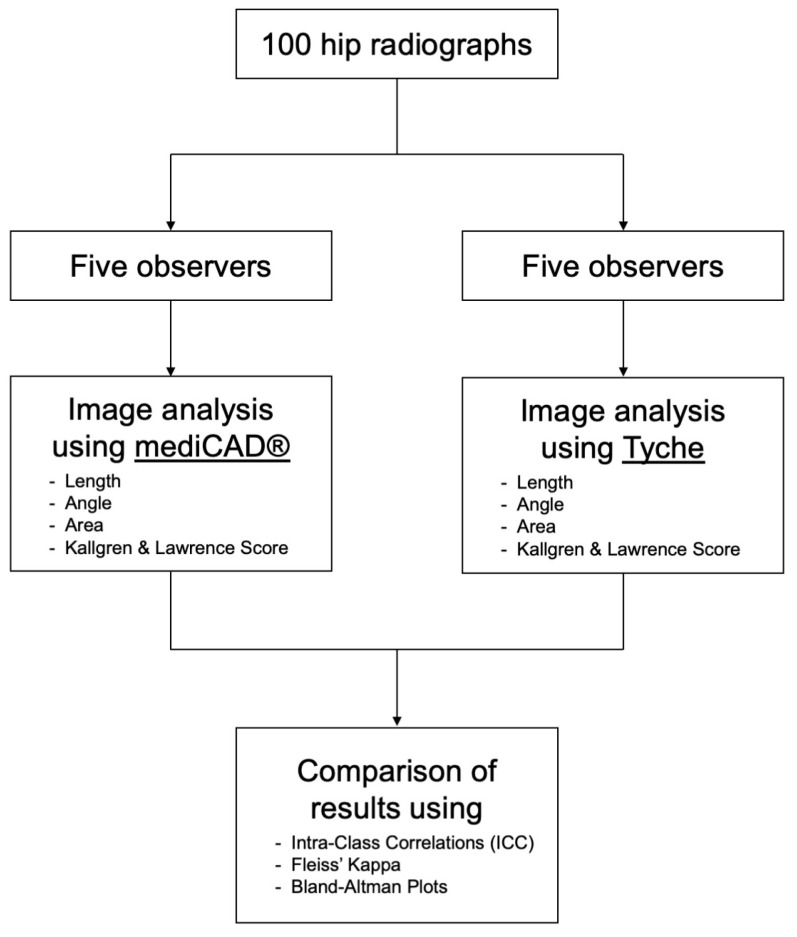
Flowchart showing the study design. In total, 100 hip radiographs were analyzed by 5 orthopedic surgeons, once using mediCAD^®^ and once using Tyche. The observers carried out length, angle, and area measurements and determined the Kallgren and Lawrence score for each radiograph. The results were compared using intra-class correlations (ICCs), Fleiss’ kappa coefficients, and Bland–Altman plots.

Figure 3 shows a flowchart of the study design.

## 3. Results

### 3.1. Measurements and Reliability Analysis

The mean results from the measurements, including the minimum (Min), maximum (Max), and standard deviation (SD) values, are shown in Table 2. Interobserver reliability was calculated between all five observers using ICCs and Fleiss’ kappa, once for the results acquired via Tyche and once for the results obtained via mediCAD^®^ (Table 3). Since every measurement was performed twice, once using Tyche and once using mediCAD^®^, the intraobserver reliability was calculated by comparing the results for each observer when using Tyche with those obtained via mediCAD^®^, using the ICCs and Fleiss’ kappa (Table 4).

**Figure 4:**
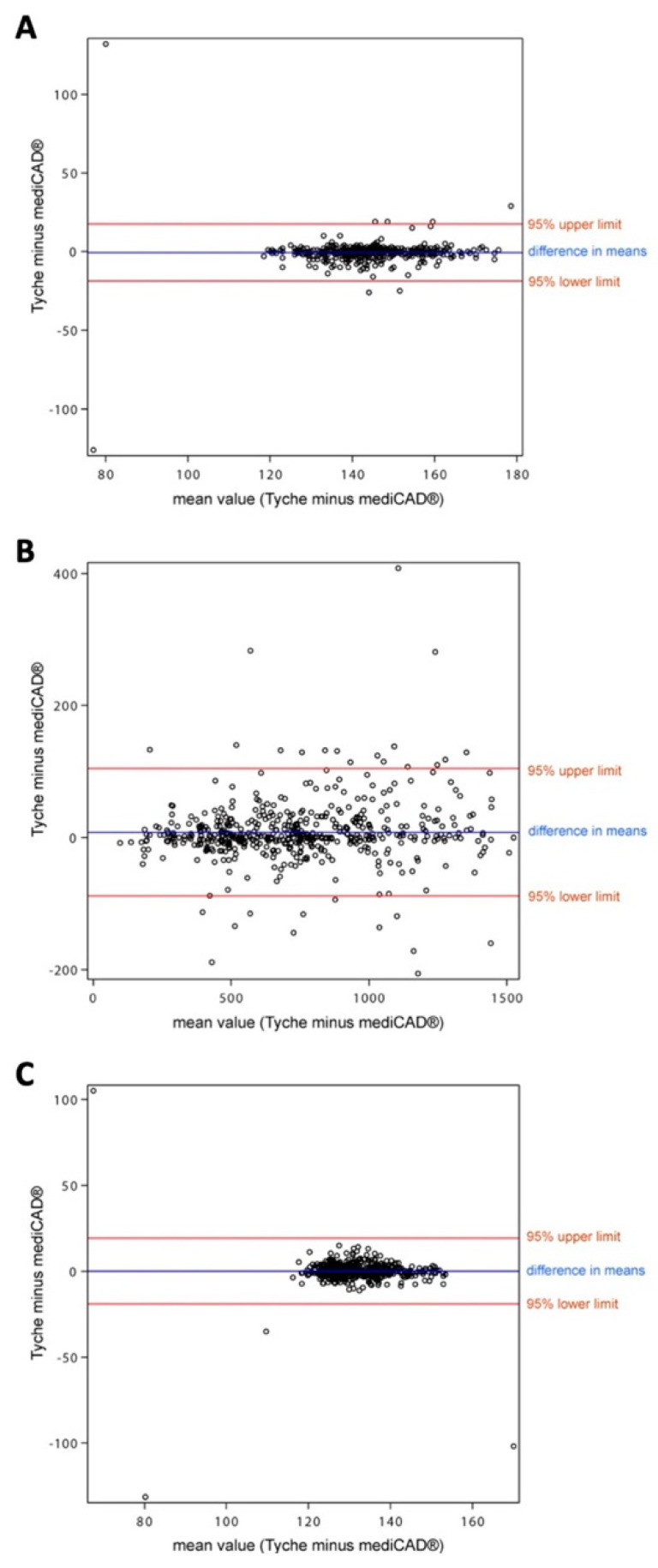
Non-inferiority of Tyche vs. mediCAD^®^ for length (**A**), area (**B**), and angle measurements (**C**), as shown via Bland–Altman plots.

Finally, Bland–Altman plots confirmed the non-inferiority of either tool by comparing the metric measurements, as shown in Figure 4.

On each Bland–Altman plot, one dot represents two measurements from the same picture, one in Tyche and one in mediCAD^®^. Differences in the measurements from Tyche vs. mediCAD^®^ (*y*-axis) are plotted against the mean values (*x*-axis). The horizontal blue line shows the mean of all differences; the two red lines show +/− 1.96 times the standard deviation of all differences. The more the values are scattered along the *x*-axis, the higher the distribution of measurement results. Ideally, most dots should be within the 95% agreement interval (red lines) on the *y*-axis, which is the case for all three plots.

### 3.2. Time

The mean time taken to analyze a single image using Tyche was 41.8 ± 2.9 s (Min–Max = 38–45), and the time taken to analyze a single image was 85.6 ± 79 s (Min–Max: 79–93) when using mediCAD^®^. Thus, the images were analyzed significantly faster when Tyche was used (paired *t*-test, *p* < 0.01).

## 4. Discussion

Correct image analysis plays a crucial role in orthopedic science and research. Blinded and collaborative assessments are important for increasing objectivity. Here, we tested a new tool for image analysis, Tyche. Tyche displays anonymized images in any web browser, with the means to store results in the same window. A collaborative approach is facilitated by inviting observers with temporarily valid project-specific URLs to perform the analysis via any web browser. The results are immediately merged and made visible to the project manager.

In this study, one hundred pelvic radiographs were analyzed by five orthopedic surgeons (four residents with at least three years of experience and one senior attending). Three different orthopedic measurement modalities (length, area, and angle), as well as a standard score (KLS), were determined using both a validated tool, mediCAD^®^, and the new tool, Tyche. Metric measurements were compared using Bland–Altman plots. As most results were found within the 95% confidence interval, the non-inferiority of either method was confirmed. Interestingly, the inter- and intraobserver correlation coefficients diverged widely, showing only a slight agreement for the KLS (Fleiss’ kappa = 0.17–0.19) value and excellent agreement for the area measurements (ICC = 0.99).

The correlation coefficients for the KLS found in this study contrast with those reported in the literature, ranging from 0.49 to 0.75 [25]. Even though the KLS is amongst the most widely used scores for assessing osteoarthritis [26], it must be noted that its poor and diverging agreement has led to the conclusion that the assessment of osteoarthritis may not be based solely upon the KLS [25,27]. Possible explanations for the relatively low agreement in this study are the high number of observers, the competence of the observers, or the use of a difficult set of images.

While the measurements of length and area show good to excellent (0.84–0.99) inter- and intraobserver agreement, the angle measurements only exhibit moderate (0.65–0.75) reliability. In this study, for angle measurements, the CCD angle was determined. Reported correlations vary between 0.62 and 0.98 [28] and are thus higher than in this study. This can have multiple explanations. Firstly, the exact definition of the CCD angle and how it should be determined are debatable [29]. Additionally, on pelvic radiographs, only a short femoral shaft is depicted. This could potentially lead to difficulty and thus inaccuracy in determining the femoral axis.

Looking at Table 4, one could also argue that the low levels of agreement for the angle measurements and the KLS are because Tyche is inferior or contains a systemic error. For example, Tyche could have an algorithm error that stores results incorrectly. Likewise, the observers could have made errors in noting their results via pen and paper when using mediCAD^®^. However, the correlation coefficients are on the same level when the results from observers using one tool were compared (Table 3). While this theoretically does not rule out the possibility of Tyche having an error or the observers having made copy-and-pasting or merging errors, it demonstrates the non-inferiority of either tool.

While the ICC and Fleiss’ Kappa coefficients show similar values for inter- and intraobserver comparisons (Table 3 vs. Table 4), the interobserver reliability and confidence intervals are slightly higher for all modalities except the area measurements. For instance, for angle measurements, the ICC is 0.75 for Tyche and 0.73 for mediCAD^®^; likewise, the confidence intervals are higher for Tyche (0.67–0.82, delta = 0.15) than mediCAD^®^ (0.64–0.81; delta = 0.17) (Table 3). Theoretically, the results acquired via Tyche are more precise. However, the difference is only marginal and does not affect the interpretation of individual coefficients. Thus, more accurate measurements with fewer errors should not be concluded, yet it at least shows that Tyche is not inferior.

In this study, it was shown that the analysis of a single image was completed significantly faster when Tyche was used. This can be explained by the streamlined workflow, which does not require opening images one by one or storing results inside separate spreadsheets. The time needed to analyze a single image was deducible from Tyche’s log files. The durations reported when mediCAD^®^ was used must be interpreted more cautiously since the observers were asked to note the time at a given point they could choose. The observers were asked to record the time required to analyze twenty images. This amount was selected as it was considered possible to analyze twenty images at a time without taking a break or experiencing distraction, yet further studies are required to scientifically prove that a streamlined workflow results in faster analysis. Furthermore, the fact that the observers could perform the analyses wherever and whenever they desired could have increased the willingness of the experts to participate in the study and shortened the time taken to complete the analysis. Again, this remains a theoretical consideration.

When Tyche was used, the results were stored inside the browser in the same window in customizable input forms. In contrast to the analysis that employed mediCAD^®^, no additional pen and paper or spreadsheets were needed. Writing down the results and merging them can be a significant source of errors. In the literature, the occurrence of errors during the transcription of data from paper into electronic forms such as spreadsheets is a well-known phenomenon with possibly significant outcomes [10,11]. Although they are difficult to investigate in real scenarios, errors occurring during the use of spreadsheet software are a problem of interest in science as they can have devastating consequences when wrong conclusions are drawn in human sciences. This problem has led to the foundation of a “European Spreadsheet Risks Interest Group” [30]. By reducing the steps in which a pen and paper or spreadsheets are needed for noting or merging results, Tyche might reduce the likelihood of transcription and calculation errors and could make results more reliable. It must be noted that this advantage remains theoretical and was not scientifically proven in this study.

In this study, all the observers worked for the same institution. Thus, the fact that Tyche allowed the observers to participate in the study online did not provide substantial benefits other than comfort. If it was desirable to include experts from different institutions, countries, or even continents, Tyche could offer a clear advantage with a streamlined workflow that only requires sharing a URL to invite experts, which could be especially helpful for multicenter studies. This advantage could further manifest in the setting of registration studies for new drugs or therapies in which images must be rigorously assessed by experts in the field from different countries. Full anonymization is of paramount importance to exclude any sort of bias or subjectivity.

The question remains whether a workflow implemented via a tool such as Tyche is necessary in an era in which automated image analyses may be conducted via machine learning and artificial intelligence (AI). In fact, Tyche requires every image to be assessed individually by observers, which limits the number of images that can be analyzed. In contrast, once an AI algorithm is set up and validated, the number of images is, theoretically, not limited. However, algorithms rely on training data and comprise a risk of bias [31,32] with increasing safety concerns [33]. For smaller studies and research projects that produced only limited amounts of imaging data, training or at least validating a machine learning algorithm could also be challenging. Thus, Tyche could be used to complement AI projects by allowing experts to efficiently train or validate algorithms. Generally speaking, Tyche could be very useful for smaller projects in which using multiple blinded observers that assess the images separately is desirable. In larger imaging projects, Tyche could be beneficial for confirming automatically generated results from AI.

This study has several limitations that need to be considered. Firstly, only a very narrow spectrum of the theoretically possible measurement modalities in orthopedics and image analysis in general were studied. In addition, apart from a probably faster analysis, no superiority of Tyche can be concluded from the study design and the present data. However, the goal was to validate the tool and therefore demonstrate its non-inferiority, which was achieved. Different study designs and broader applications are needed to evaluate whether Tyche provides any superiority.

## 5. Conclusions

We conclude that Tyche is a valid tool for conducting blinded and collaborative orthopedic image assessments which could increase the objectivity of results and make conducting studies more efficient. Tyche can be used for inter- and intraobserver analyses, score validations, and multicenter studies.

## Figures and Tables

**Table 1:** Interpretations for intra-class correlation and Fleiss’ kappa coefficients.

ICC	Interpretation	Fleiss’ Kappa	Interpretation
>0.90	excellent	>0.81	almost perfect
>0.75	good	>0.61	substantial
>0.50	moderate	>0.41	moderate
≤0.50	poor	>0.21	fair
		>0.01	slight

ICC = intra-class correlation.

**Table 2:** Results of metric measurements from mediCAD^®^ and Tyche.

		mediCAD^®^	Tyche
Length [mm]	Mean ± SD	143.4 ± 10.9	144.1 ± 10.5
	Min–Max	117.4–172.8	119.2–174.0
Area [mm^2^]	Mean ± SD	745.9 ± 298.3	737.8 ± 294.5
	Min–Max	203.8–1444.8	212.6–1446.4
Angle [degrees]	Mean ± SD	132.3 ± 6.1	132.1 ± 6.5
	Min–Max	113.5–145.9	102.2–146.5

SD = standard deviation, Min = minimum, Max = maximum.

**Table 3:** Interobserver reliability for Tyche and mediCAD^®^.

	Length	Angle	Area	KLS
	ICC (CI)			Fleiss’ Kappa (CI)
Tyche	0.93 (0.91–0.95) *	0.75 (0.67–0.82) *	0,99 (0.99–0.99) *	0.19 (0.15–0.22) *
mediCAD^®^	0.92 (0.89–0.94) *	0.73 (0.64–0.81) *	0.99 (0.99–0.99) *	0.18 (0.14–0.21) *

Five observers determined three metric variables (length, angle, and area) and the Kallgren and Lawrence score on one hundred hip radiographs, once while using Tyche and once while using mediCAD^®^. The interobserver reliability was calculated between all five observers, once for the results from Tyche and once for the results from mediCAD^®^. Except for area measurements, the results from Tyche demonstrate slightly higher ICC and Fleiss’ Kappa coefficients and higher confidence intervals with lower deltas. ICC = intra-class correlation coefficient; CI = confidence interval; KLS = Kellgren and Lawrence score. * = *p*-value < 0.001.

**Table 4:** Intraobserver reliability: Tyche versus mediCAD^®^.

	Length	Angle	Area	KLS
	ICC (CI)			Fleiss’ Kappa (CI)
Tyche vs. mediCAD^®^	0.84 (0.81–0.87) *	0.65 (0.59–0.71) *	0.99 (0.99–0.99) *	0.17 (0.12–0.22) *

Five observers determined three metric variables (length, angle, and area) and the Kallgren and Lawrence score on one hundred hip radiographs, once while using Tyche and once while using mediCAD^®^. The results from Tyche were compared with those from mediCAD^®^. Intraobserver reliability was excellent for area measurements and slight for the K and L score. ICC = intra-class correlation coefficient; CI = confidence interval; KLS = Kellgren and Lawrence score. * = *p*-value < 0.001.

## Data Availability

Data are available upon request due to privacy restrictions.
